# Hepatocellular carcinoma systemic treatment update: From early to advanced stage

**DOI:** 10.1016/j.bj.2024.100815

**Published:** 2024-11-17

**Authors:** Wei Teng, Tai-Chi Wu, Shi-Ming Lin

**Affiliations:** a Department of Gastroenterology and Hepatology, Chang Gung Memorial Hospital, Linkou Medical Center, Taoyuan, Taiwan; b College of Medicine, Chang Gung University, Taoyuan, Taiwan; c Liver Research Center, Chang Gung Memorial Hospital, Linkou Medical Center, Taoyuan, Taiwan

**Keywords:** Hepatocellular carcinoma, Tyrosine kinase inhibitors, Immune checkpoint inhibitors, Neoadjuvant therapy

## Abstract

Hepatocellular carcinoma (HCC) ranks the sixth most common malignancy but the third leading cause of cancer-related mortality in the world. Significant breakthroughs have been made in systemic treatment for HCC over the past two decades, which have improved treatment outcomes. In addition to multiple tyrosine kinase inhibitors (mTKIs), immune checkpoint inhibitors (ICIs) and antiangiogenic drugs are increasingly being applied. The combination of ICI and antiangiogenic or dual ICIs has become the new standard of care due to remarkable response rates. However, currently available systemic regimens are primarily reserved for certain patients in the intermediate and advanced stages who will not benefit from locoregional treatments. Evidence supporting the use of systemic treatment as neoadjuvant or adjuvant therapies in patients with early-stage HCC, especially the high risk of recurrence after curative treatments, remains limited. This review identified recent developments in systemic therapy, including mTKIs and ICIs, considering results on first- and second-line treatment, role of neoadjuvant and adjuvant settings, and combination with loco-regional therapy. Various ongoing clinical trials regarding the role of systemic therapies and potential novel targets in patients with early-, intermediate-, and advanced-stage HCC were also summarized and revealed that systemic therapy is no longer limited to advanced-stage HCC. Moreover, the introduction of T-cell redirecting strategies, including bispecific antibodies and chimeric antigen receptor T cells, has revolutionized the treatment landscape for HCC. Future research should focus on an in-depth exploration of the mechanisms governing the establishment of tumor barriers.

## 1 Introduction

Hepatocellular carcinoma (HCC) is the sixth most common cancer and the third leading cause of cancer-related deaths worldwide [1]. Viral infections, including chronic hepatitis B and hepatitis C, as well as non-alcoholic fatty liver disease can cause HCC [2]. Although the improvements of hepatitis control through vaccination and antiviral therapy, HCC remains a critical public health concern. We have experienced great advances in the systemic treatment including four oral multi-tyrosine kinase inhibitors (mTKIs) (sorafenib, lenvatinib, regorafenib, and cabozantinib), two antiangiogenic antibody (ramucirumab and bevacizumab), and six immune checkpoint inhibitors (ICIs) for administration alone or in combination (atezolizumab in combination with bevacizumab, tremelimumab in combination with durvalumab, ipilimumab in combination with nivolumab, and pembrolizumab monotherapy) of HCC over the past two decades. The evolution of systemic therapies has given great improvement of the prognosis for patients with unresectable HCC who are unsuitable for locoregional therapy, including patients with intermediate-stage HCC who will not benefit from transarterial chemoembolization (TACE), such as infiltrative, diffuse, and extensive bilobar involvement, and those with advanced-stage HCC [3,4]. Atezolizumab plus bevacizumab and tremelimumab plus durvalumab are currently considered as first-line treatments for patients in the advanced stages, with no contraindications in the global clinical guidelines [[5], [6], [7], [8], [9], [10], [11]].

Despite an inspiring median overall survival (OS) over 60 months after resection or local ablation, the recurrence rate is estimated to be approximately 70% and 20% at 5 years after liver resection and after liver transplantation, respectively, for patients with early-stage HCC [12,13]. Studies on neoadjuvant and adjuvant systemic therapies to prevent recurrence in these patients, particularly those with a high risk of tumor recurrence before treatment, are limited. In this review, we identified recent developments in systemic therapy, including mTKIs and ICIs, considering results on first- and second-line treatment, role of neoadjuvant and adjuvant settings, and combination with loco-regional therapy. We also summarized various ongoing clinical trials regarding the role of systemic therapies and potential novel targets in patients with early-, intermediate-, and advanced-stage HCC.

## 2 Emerging agents for systemic HCC treatment

Currently approved systemic therapies are broadly categorized into two groups: (1) antiangiogenic targeted therapies and (2) ICIs. Antiangiogenic targeted therapies include mTKIs (sorafenib, lenvatinib, cabozantinib, and regorafenib) and monoclonal antiangiogenic antibodies (ramucirumab and bevacizumab). ICIs currently include inhibitors of programmed death 1 (PD-1) (pembrolizumab and nivolumab), programmed death ligand 1 (PD-L1) (durvalumab and atezolizumab), and cytotoxic T lymphocyte-associated protein 4 (CTLA-4) (tremelimumab and ipilimumab).

## 3 Management of early-stage HCC

Resection is considered as the standard therapy for patients with or without cirrhosis with well-preserved liver function, solitary tumors and clinically mild portal hypertension (hepatic vein to portal system gradient ≤10 mmHg) [14], and liver transplantation (LT) is indicated for patients who are not suitable for resection who meet the Milan criteria (single tumor ≤5 cm or multiple tumors as ≤ 3 nodules size ≤3 cm) without vascular invasion and/or extrahepatic involvement [15] or after adequate downstaging of the tumor [16]. Thermal ablation with radiofrequency ablation or microwaves offers an alternative modality as curative therapy for small tumors not amenable to resection or transplantation, providing excellent outcomes with minimally invasive procedures [17].

### 3.1 Risk factors of tumor recurrence

Nevertheless, the cumulative 5-year recurrence rate after operation or local ablation remains high around 50%–70%, especially during the first 12 months after curative treatment [18]. The early peak of recurrence was approximately 1 year postoperatively while the second peak was around after 4 postoperative years [19]. These early recurrences were usually due to occult metastases far distal from the primary resection margin [20,21], which were presumed to be already present at the time of resection. On the contrary, the majority of the second peak was attribute to new lesions (de novo HCCs) related to the underlying liver disease [22]. The two types of recurrence are two distinct entities associated with different risk factors. The factors affecting the possibility of early HCC relapse can be classified into three categories: those related to the tumor such as size, number of nodules, differentiation, and oncological markers; to the patient such as age, comorbidity, liver function, possible viral load, presence and activity of hepatitis, presence and activity of liver cirrhosis; treatment such as type of treatment, margins, and characteristics of resection [23]. Conversely, late recurrence does not depend on the characteristics of the previous tumor. About 90% of late relapses consist in exclusively intrahepatic localization, whereas the remaining cases show both intrahepatic and extrahepatic localizations. Therefore the risk factors are mainly related to underlying liver disease such as age, gender, etiology and cirrhosis [22].

### 3.2 Tertiary prevention of tumor recurrence by antiviral therapy

Antiviral therapies could maximize long-term inhibition of virus replication, reduce liver damage caused by the virus, prevent disease progression, provide a good basis for liver function, reduce the risk of recurrence, and ultimately prolong patient survival in both chronic hepatitis B virus or hepatitis C virus infected patients [13]. In chronic hepatitis B patients, nucleos(t)ide analogues (Nuc) provide a more effective HCC tertiary prevention effect than an interferon (IFN)-based regimen. In chronic hepatitis C patients, the tertiary prevention effect by direct acting antiviral agents (DAAs) was reported non-inferior to that by IFN-based therapy. Therefore, antiviral therapy is crucial for preventing postoperative recurrence of HBV- or HCV-related HCC.

### 3.3 Neoadjuvant therapies might outperform adjuvant therapies

The delivery of treatment before surgery has several important goals. First, nonvisible or micro-metastatic tumors may be eradicated, reducing the early recurrences within 2 years of resection in HCC [18]. Second, treatment before resection may reduce the extent of required surgical resection, which may conserve liver function thus improving outcomes. Downstaging to resectability can further expand the number of patients who can benefit from curative approaches. Third, neoadjuvant approaches can provide prognostic information with increasing recognition of major pathologic response (MPR) as both <10% and <30% viable cells or “significant necrosis rates” for predicting OS [24]. Adjuvant ICIs stimulate antitumor immunity against micro-metastases after the primary tumor is removed, whereas neoadjuvant immunotherapies use the primary tumor as a source of antigens to stimulate such responses; in both situations, those micro-metastases can eventually lead to disease recurrence. Antitumor immune responses with immunotherapy depend on interactions among T cells, antigen-presenting cells and tumor cells. Such interactions are more likely to occur when a large burden of primary tumor (containing the antigens targeted by the immune system) is still present, providing a potential mechanistic rationale for why neoadjuvant immunotherapies might be preferable to adjuvant immunotherapies [25].

Among the published results of neoadjuvant immunotherapy in HCC, one phase I trial tried to analyze the efficacy of combination of nivolumab plus cabozantinib for 3 months in 15 enrolled patients with the aim of curative-intent resection [26]. Overall, twelve patients successfully underwent resection and five encountered a major pathological response with definition as ≥ 90% tumor necrosis. Another resemble phase II study assessed the effects of neoadjuvant cemiplimab (anti-PD-L1 antibody) in patients considered as candidates for resection [27]. These patients received two doses of cemiplimab and underwent resection as early as three weeks after starting therapy, then they received up to eight additional cycles of cemiplimab. Among the 20 patients who underwent resection, 20% of them had significant tumor necrosis. In another phase II study, patients received nivolumab with or without a single dose of ipilimumab for 6 weeks before resection and thereafter up to 2 years postoperatively [28]. Significant tumor necrosis occurred in 33% of 9 patients treated with nivolumab monotherapy and in 27% of 11 patients treated with nivolumab plus ipilimumab. Despite the small sample sizes of these trials, the study of nivolumab plus ipilimumab demonstrated that ipilimumab was markedly effective in patients with cold tumors, supporting the theory that CTLA-4 blockade might be the most beneficial in patients lacking pre-existing anti-tumor immunity.

The current ongoing study included the DYNAMIC trial, a phase II study (NCT04954339) investigating the efficacy of neoadjuvant atezolizumab plus bevacizumab in patients with potentially resectable or high-risk resectable HCC, and AB-LATE02, a phase II study (NCT04727307) assessing the efficacy of a combination of atezolizumab monotherapy before and atezolizumab plus bevacizumab after radiofrequency ablation. Other active phase II trials of ICI-based neoadjuvant therapy in early stages are listed in [Table 1].

**Table 1:** Summary of active clinical phase II trials of immune checkpoint inhibitor-based neoadjuvant therapy in the early stage HCC.

Trial name (Registration No.)	Characteristics		End-point	Key findings
	Regimen	Arm		
DYNAMIC (NCT04954339)	Atezolizumab plus bevacizumab	Two cycles of atezolizumab plus bevacizumab prior to surgical resection and four cycles of atezolizumab plus bevacizumab after the surgery will be administered	pCR, AE, PFS, RFS	–
AB-LATE02 (NCT04727307)	Atezolizumab plus bevacizumab	Neoadjuvant atezolizumab and adjuvant atezolizumab plus bevacizumab in combination with percutaneous radiofrequency ablation	RFS	–
ADVANCE HCC (NCT05137899)	Atezolizumab plus bevacizumab	**Arm 1**: Neoadjuvant atezolizumab and bevacizumab 4 cycles prior to surgical resection **Arm 2**: Neoadjuvant stereotactic body radiation therapy (SBRT) prior to surgical resection	Resection, ORR, AE, PFS, OS	–
NEOTOMA (NCT05440864)	Tremelimumab plus durvalumab	Tremelimumab plus durvalumab preoperatively, followed by adjuvant durvalumab	AE, ORR, pCR, LR, RFS, OS, biomarkers	–
NIVOLEP (NCT03630640)	Nivolumab	Nivolumab 240 mg Q2W neoadjuvant prior to electroporation then nivolumab 480 mg Q4W adjuvant for 12 months	RFS, ORR, OS, AE	Radiological assessment (n = 19): Reduction in 29% of all HCC nodules and 68% of nodules were stable Pathological response (n = 17): Tumor response in 17% and an isolated increase of peritumoral and intratumoral infiltrating lymphocytes demonstrated in 23% Any grade AE: 0%
NeoLEAP-HCC (NCT05389527)	Pembrolizumab plus lenvatinib	Pembrolizumab 200 mg and lenvatinib for 9 weeks prior to resection. Pembrolizumab and lenvatinib will restart as adjuvant treatment for up to 1 year after resection.	MPR, pCR, ORR, R0 resection	Pathological response (n = 37): 37.8% MPR, 8.1% pCR, 29.7% significant tumor necrosis Any grade AE: 74.4% Grade 3 AE: 14.0%
PRIMER-1 (NCT05185739)	Pembrolizumab plus lenvatinib	**Arm 1**: Pre-operative pembrolizumab (200 mg) for 2 cycles **Arm 2**: Pre-operative lenvatinib for 6 weeks **Arm 3**: Pre-operative combination of pembrolizumab and lenvatinib at the standard doses and duration	MPR, ORR, RFS, AE	–
TALENT (NCT04615143)	Tislelizumab plus lenvatinib	**Arm 1**: Pre-operative tislelizumab for 2 cycles then tislelizumab for 1 year in every 4–6 weeks after surgery **Arm 2**: Pre-operative tislelizumab for 2 cycles and lenvatinib for 4 weeks then tislelizumab plus lenvatinib for 1 year in every 4–6 weeks after surgery	DFS, ORR, AE, MPR	**Arm 1 (n** = **11):** ORR: 18.2% Pathological response: 2 tumor necrosis over 70% and the others pathologic response of 5–35% Any grade AE: 36.4%

Abbreviations: AE: adverse events; DFS: disease free survival; MPR: Major Pathological Response; ORR: objective response rate; OS: overall survival; pCR: Pathological Complete Response; PFS: progression-free survival; RFS: Recurrence-Free Survival.

### 3.4 The role of adjuvant therapies for early-stage HCC

The absolute benefit of adjuvant therapies relate directly to the risk of recurrence, which for HCC can be broadly grouped into features that reflect the aggressive tumor and the underlying liver disease. Besides, data from a landmark study [29] using an orthotopic model of breast cancer showed that delivering ICIs after surgery enabled T cell expansion, and yielded the best antitumor activity while also decreasing toxicity. The prevention or delay of HCC recurrence after hepatic resection or local ablation with adjuvant therapies was an unmet medical need. Several randomized controlled trials have assessed the effect of adjuvant therapies on recurrence-free survival (RFS) after curative treatments in the past decades [30]. However, most of the studies failed to reach primary endpoint and validation of the positive studies is still pending. For example, the adjuvant use of retinoids [31], vitamin K2 [32], or interferon-α [33] and ^131^I-lipiodol embolization [34] failed to show significant efficacy. Likewise, the phase III STORM trial that treated with adjuvant sorafenib after resection or local ablation did not show an improvement in RFS compared with placebo [35]. Sirolimus, a mTOR inhibitor, did not improve RFS in patients with HCC who underwent liver transplantation in the SiLVER trial [36]. Recently, results from the phase III IMbrave050 trial have shown that patients at high risk of recurrence after resection or local ablation who received adjuvant 1-year atezolizumab plus bevacizumab therapy compared with only active surveillance have significant improvement of RFS [37]. Within the median follow-up duration of 17.4 months, the statistical analyses favored adjuvant atezolizumab plus bevacizumab over active surveillance (HR, 0.72; 95% CI, 0.56–0.93; p = 0.012) although the median RFS was not reached in each group. The respective incidence of grade 3-4 adverse events (AEs) was 41% and 13% in atezolizumab plus bevacizumab and active surveillance, and 9% of patients withdrew from atezolizumab and bevacizumab due to AEs. Furthermore, 8.4% versus 1% of patients encountered immune-related AEs that required systemic corticosteroids. However, the initial RFS benefit with atezolizumab plus bevacizumab versus active surveillance was not sustained during the subsequent updated analysis that did not support these combine therapy as an adjuvant therapy for all high-risk HCC.

Another one phase II SORAMIC trial [38] enrolled patients randomized equally to local ablation plus sorafenib versus local ablation plus placebo. However, adjuvant sorafenib did not improve the time-to-recurrence or local control rate after local ablation in patients with HCC within the limitations of an early terminated trial. Multiple research are now investigating additional post-curative treatments in patients with early-stage HCC [Table 2], including the following: 1) the phase III trial EMERALD-2 (NCT03847428) [39], evaluating the efficacy and safety of durvalumab monotherapy and durvalumab combined with bevacizumab as adjuvant therapy in patients with HCC after curative resection or ablation who are at a high risk of recurrence; 2) the phase III trial CheckMate 9DX (NCT03383458) [40], evaluating the impact of nivolumab on RFS in patients with HCC after resection or ablation who are at a high risk of recurrence; and 3) the phase III trial KEYNOTE-937 (NCT03867084) [41], evaluating the efficacy and safety of pembrolizumab versus placebo after surgical resection or local ablation. Future studies with large sample size are needed to validate the advantages of adjuvant therapy in the aim of improvement of survival benefits compared with the results found from IMbrave050 trial.

**Table 2:** Summary of active clinical trials of immune checkpoint inhibitor-based adjuvant therapy in the early stage HCC.

Trial name (Registration No.)	Characteristics			End-point	Key findings
	Phase	Regimen	Arm		
AB-LATE02 (NCT04727307)	II	Atezolizumab plus bevacizumab	Neoadjuvant atezolizumab and adjuvant atezolizumab plus bevacizumab in combination with percutaneous radiofrequency ablation	RFS	–
IMbrave050 (NCT04102098)	III	Atezolizumab plus bevacizumab	**Arm 1**: Atezolizumab plus bevacizumab after resection or ablation (n = 334) **Arm 2**: Active surveillance (n = 334)	RFS, OS, TTR, AE	RFS: HR 0.72; 95%CI: 0.56–0.93; p = 0.012 Any grade AE: 98% vs. 62% Grade 3/4 AE: 41% vs. 13%
EMPHASIS (NCT05516628)	II	Atezolizumab plus bevacizumab	Adjuvant atezolizumab plus bevacizumab for a year following surgery	RFS, TTR, OS	–
EMERALD-2 (NCT03847428)	III	Durvalumab plus bevacizumab	**Arm 1**: Durvalumab plus bevacizumab after resection or ablation **Arm 2**: Durvalumab monotherapy after resection or ablation **Arm 3**: Placebo after resection or ablation	RFS, OS, TTR	–
CheckMate 9DX (NCT03383458)	III	Nivolumab	**Arm 1:** Nivolumab after curative resection or ablation **Arm 2:** Placebo after resection or ablation	RFS, OS, TTR	–
NIVOLEP (NCT03630640)	II	Nivolumab	Nivolumab 240 mg Q2W neoadjuvant prior to electroporation then nivolumab 480 mg Q4W adjuvant for 12 months	RFS, ORR, OS, AE	Radiological assessment (n = 19): Reduction in 29% of all HCC nodules and 68% of nodules were stable Pathological response (n = 17): Tumor response in 17% and an isolated increase of peritumoral and intra-tumoral infiltrating lymphocytes demonstrated in 23% Any grade AE: 0%
KEYNOTE-937 (NCT03867084)	III	Pembrolizumab	**Arm 1**: Pembrolizumab after resection or ablation **Arm 2**: Placebo after resection or ablation	RFS, OS, AE, QoL	–
JUPITER-04 (NCT03859128)	III	Toripalimab	**Arm 1**: Toripalimab after resection **Arm 2**: Placebo after resection	RFS, TTR, OS, AE	–

Abbreviations: AE: adverse events; DFS: disease free survival; ORR: objective response rate; OS: overall survival; PFS: progression-free survival; QoL: quality of life; RFS: Recurrence-Free Survival; TTR: Time-to-Response.

## 4 Management of intermediate-stage HCC

### 4.1 TACE plus mTKIs

TACE remains the standard treatment for patients with intermediate-stage HCC. However, those with confluent multinodular tumors, simple nodular tumors with extranodular growth, diffuse as well as massive type tumors might be considered unsuitable for TACE [[42], [43], [44]]. Therefore, they are considered as candidates for systemic therapies, given the low probability of objective responses rate (ORR) and the high risk of liver injury after embolic therapies. mTKIs could provide antitumor effects, normalize vessel system in the tumor microenvironment [[45], [46], [47]], and suppress the release of vascular endothelial growth factor post embolic therapies [48]. The concurrent add of mTKIs can therefore strengthen the efficacy of TACE and inhibit the proliferation of residual tumors as well as new onset of other intrahepatic lesions. Several clinical trials [[49], [50], [51], [52], [53]] have been conducted based on these concepts, but the results are not encouraging except two trials including the TACTICS [54] and LAUNCH [55] trials. The TACTICS was a randomized, phase II study that compared TACE plus sorafenib with TACE alone. The OS of TACE plus sorafenib did not show significant benefit over TACE alone (HR, 0.861; 95% CI, 0.607–1.223; *p* = 0.40). However, the progression-free survival (PFS) of combination group demonstrated better result than the TACE alone group (HR, 0.661; 95% CI, 0.466–0.938; *p* = 0.02). Furthermore, patients with tumor burden beyond up-to-seven criteria who were treated with TACE plus sorafenib had better both PFS and OS (PFS: 22.1 vs. 9.0 months; OS: 36.3 vs. 25.0 months). The LAUNCH study was a randomized, phase III trial that compared lenvatinib plus TACE (LEN-TACE group) and lenvatinib monotherapy (LEN group). The LEN-TACE group had not only longer OS (17.8 vs. 11.5 months; HR, 0.45; *p* < 0.001) but also PFS (10.6 vs. 6.4 months; HR, 0.43; *p* < 0.001) than the LEN group. Patients in the LEN-TACE group also had a better ORR than those in the LEN group, according to mRECIST (54.1% vs. 25.0%; *p* < 0.001). Recently, the single-arm, phase II TACTICS-L trial [56] recruited 62 patients receiving lenvatinib plus TACE revealed the best ORR with 88.7% and complete response rate with 67.7% by RECICL, respectively. The primary endpoint of the median PFS was 28.0 months, and the secondary endpoint of the median OS was not achieved, indicating the efficacy of LEN-TACE in patients who are ineligible for locoregional therapy in the future.

### 4.2 TACE plus ICIs

Several studies are currently underway to assess the efficacy and safety of combining TACE with the systemic therapy of ICI and antiangiogenic drugs. The inspiring response rates to atezolizumab plus bevacizumab raise the question of which patients can be expected to benefit from combination therapy, even at the intermediate stage. The potential benefits of systemic therapy versus TACE need to be reevaluated especially for patients whom are at high risk of recurrence after TACE. Two atezolizumab plus bevacizumab-based trials have been reported: the phase II DEMAND trial (NCT04224636) [57], the first trial to evaluate the safety and efficacy of atezolizumab plus bevacizumab before or in combination with TACE in patients with intermediate-stage HCC in terms of the primary endpoint of the 24-month survival rate and the secondary endpoints of ORR, PFS, and safety and quality of life; the phase III TALENT-ACE trial (NCT04712643) [58], which investigated atezolizumab plus bevacizumab in combination with TACE in patients with unresectable HCC. Two other ongoing trials included the following: the phase III ABC-HCC trial (NCT04803994) [59], investigating atezolizumab plus bevacizumab versus TACE in intermediate-stage HCC with a high tumor burden exceeding the Milan criteria; and the phase II LOST-B trial (NCT05537402), evaluating the efficacy and safety of atezolizumab plus bevacizumab compared with locoregional therapy (TACE or transarterial radioembolization).

When considering durvalumab-based therapy, the phase III EMERALD-1 trial (NCT03778957) [60] investigated the efficacy and safety of TACE in combination with durvalumab monotherapy or durvalumab plus bevacizumab versus TACE alone. The primary endpoint was met with a significant longer median PFS in the durvalumab plus bevacizumab plus TACE group compared with TACE alone (15.0 vs. 8.2 months; HR, 0.77; 95% CI, 0.61–0.98; *p* = 0.032). Considering the significant increase in PFS in patients with intermediate-stage HCC in the EMERALD-1 trial, the use of durvalumab plus bevacizumab after TACE may become a standard of care in the near future. Another phase III EMERALD-3 trial (NCT05301842) [61] compared the outcomes of combining TACE with the Single Tremelimumab Regular Interval Durvalumab (STRIDE) regimen with and without lenvatinib versus TACE alone. The phase II PETAL trial (NCT03397654) [62] is the first early-phase clinical trial to test the hypothesis of synergy between TACE and sequential PD-1 targeted immunotherapy (pembrolizumab) for liver-confined HCC. The results showed that pembrolizumab yielded no synergistic or dose-limiting toxicities after TACE; the ORR at 12 weeks after TACE was 53%. The PFS rate at 12 weeks was 93%, and the median PFS was 8.95 months, encouraging further clinical development of immunotherapy alongside TACE.

Other randomized phase III clinical trials of ICI-based therapy plus TACE are ongoing now including [Table 3]: LEAP-012 (NCT04246177) [63], investigating pembrolizumab and lenvatinib in combination with TACE; and CheckMate 74W (NCT04340193) [64], evaluating the efficacy of nivolumab plus ipilimumab in combination with TACE.

**Table 3:** Summary of active clinical trials of involving TACE and immune checkpoint inhibitor-based therapy in the intermediate stage HCC.

Trial name (Registration No.)	Characteristics			End-point	Key findings
	Phase	Regimen	Arm		
ABC-HCC (NCT04803994)	III	Atezolizumab plus bevacizumab	**Arm 1**: Atezolizumab plus bevacizumab **Arm 2**: TACE and additional on demand TACE	OS, ORR, TTP, PFS, DOR, AE, QoL	–
TALENT-ACE (NCT04712643)	III	Atezolizumab plus bevacizumab	**Arm 1**: Atezolizumab plus bevacizumab plus on-demand TACE **Arm 2**: TACE alone	PFS, time to unTACEable progression, ORR, DOR, AE	–
DEMAND (NCT04224636)	II	Atezolizumab plus bevacizumab	**Arm 1**: Up-front atezolizumab plus bevacizumab, then on demand TACE **Arm 2**: Atezolizumab plus bevacizumab combined with TACE	OS, PFS, ORR, DCR, AE, QoL	–
LOST-B (NCT05537402)	II	Atezolizumab plus bevacizumab	**Arm 1**: Atezolizumab plus bevacizumab **Arm 2**: TACE or TARE alone	PFS, ORR, OS, QoL	–
EMERALD-1 (NCT03778957)	III	Durvalumab, bevacizumab	**Arm 1**: TACE followed by durvalumab (n = 207) **Arm 2**: TACE followed by durvalumab plus bevacizumab (n = 204) **Arm 3**: TACE followed by placebo (n = 205)	PFS, ORR, safety, QoL	PFS: Arm 2 vs. Arm 3: 15.0 vs. 8.2 mo (HR, 0.77; 95%CI, 0.61–0.98; p = 0.032) Arm 1 vs. Arm 3: 10.0 vs. 8.2 mo (HR, 0.94; 95%CI, 0.75–1.19; p = 0.638) ORR: 41.0% vs. 43.6% vs. 29.6% TTP: 11.5 vs. 22.0 vs. 10.0 mo Any AE: 92.7% vs. 98.1% vs. 93.0% Grade 3/4 AE: 27.6% vs. 45.5% vs. 23.0%
EMERALD-3 (NCT05301842)	III	Tremelimumab, durvalumab and lenvatinib	**Arm 1**: Tremelimumab plus durvalumab and lenvatinib with TACE **Arm 2**: Tremelimumab plus durvalumab with TACE **Arm 3**: TACE alone	PFS, OS	–
TACE-3 (NCT04268888)	III	Nivolumab	**Arm 1**: Nivolumab plus TACE **Arm 2**: TACE	OS, time to unTACEable progression, PFS, ORR, QoL	–
RENOTACE (NCT04777851)	III	Regorafenib plus nivolumab	**Arm 1**: Regorafenib plus nivolumab **Arm 2**: TACE	PFS, OS, ORR, time to unTACEable progression, DOR	–
CheckMate 74W (NCT04340193)	III	Nivolumab, ipilimumab	**Arm 1**: Nivolumab plus ipilimumab plus TACE **Arm 2**: Nivolumab plus TACE **Arm 3**: TACE alone	TTTP, OS, PFS	–
LEAP-012 (NCT04246177)	III	Lenvatinib plus pembrolizumab	**Arm 1**: Lenvatinib plus pembrolizumab plus TACE **Arm 2**: TACE alone	PFS, ORR, DCR, DOR, TTP	–
REPLACE (NCT04777851)	III	Regorafenib plus pembrolizumab	**Arm 1**: Regorafenib plus pembrolizumab **Arm 2**: TACE or TARE alone	PFS, OS, ORR, time to unTACEable progression, DOR	–
PETAL (NCT03397654)	II	Pembrolizumab	TACE followed by pembrolizumab	Safety, PFS	–

Abbreviations: AE: adverse events; DCR: disease control rate; DOR: duration of response; DFS: disease free survival; ORR: objective response rate; OS: overall survival; PFS: progression-free survival; RFS: Recurrence-Free Survival; TTP: Time-to-Progression; TTR: Time-to-Response.

## 5 Management of advanced-stage HCC

### 5.1 First-line therapies

Atezolizumab plus bevacizumab [65], durvalumab plus tremelimumab [66], lenvatinib [67], and sorafenib [68] are worldwide well-established as first-line treatments for advanced-stage HCC. Atezolizumab plus bevacizumab is further advised as the preferred first-line treatment on the basis of several global treatment guidelines [[5], [6], [7], [8], [9],^11^,^44^,^69^] if no bleeding risk, autoimmune disease or liver transplantation history are present. After a median follow-up period of 15.6 months, atezolizumab plus bevacizumab demonstrated significantly longer median OS (19.2 vs. 13.4 months; HR, 0.66; 95% CI, 0.52–0.85; *p* < 0.001) and PFS (6.9 vs. 4.3 months; HR, 0.65; 95% CI, 0.53–0.81; *p* < 0.001) than sorafenib in the IMbrave150 trial [70]. Besides, the ORR was 30% (95% CI, 25–35) in patients treated with atezolizumab plus bevacizumab higher than patients treated with sorafenib (11%, 95% CI, 7–17), according to RECIST 1.1. Apart from the benefit of atezolizumab plus bevacizumab treatment compared to sorafenib, the analyses of patient-reported outcome from the IMbrave150 trial revealed clinically significant benefits in terms of quality of life, functioning, and disease symptoms for the atezolizumab plus bevacizumab treatment [71]. Another advised first-line treatment is durvalumab plus tremelimumab. Results from the phase III HIMALAYA trial [66] revealed that durvalumab plus tremelimumab (STRIDE) provided a better overall survival benefit compared with sorafenib (16.4 vs. 13.8 months; HR, 0.78; 96.02% CI, 0.65–0.93; *p* < 0.0035) and there is no significant difference of OS between durvalumab monotherapy and sorafenib when used as first-line theraypy (16.6 vs. 13.8 months; HR, 0.86; 95.67% CI, 0.73–1.03; non-inferiority margin, 1.08). The median PFS was not significantly different among the three groups. Therefore, tremelimumab plus durvalumab is another preferred first-line option for patients who are not suitable for anti-vascular endothelial growth factor therapy, such as high risk of bleeding. Otherwise, sorafenib and lenvatinib could be considered if contraindications for immunotherapy.

In recent one first-line phase III RATIONALE 301 trial, tislelizumab (a PD-1 blockade) revealed similar OS to that with sorafenib (15.9 vs. 14.1 months; HR 0.85; *p* = 0.04); it also showed better ORR (14.3% vs. 5.4% per RECIST 1.1), longer durable responses (36.1 vs. 11.0 months), and longer median PFS (3.4 vs. 2.1 months) than sorafenib [72]. The phase III ORIENT-32 trial with sintilimab plus one bevacizumab biosimilar (IBI305), another one combination of antiangiogenic regimen and anti-PD-1/PD-L1 antibody, demonstrated both better overall survival (HR, 0.57; *p* < 0.0001) and progression-free survival (HR, 0.56; *p* < 0.0001) benefits [73] as well as improvement in ORR, time-to-recurrence, duration of response, and depth of response compared with sorafenib in the updated data [74]. The cabozantinib plus atezolizumab in the phase III COSMIC-312 trial [75] revealed superior PFS (HR, 0.63) but no statistically significant OS benefit than sorafenib. The phase III LEAP-002 study determining the efficacy of lenvatinib plus pembrolizumab did not show significantly superior OS and PFS than those treated with lenvatinib monotherapy [76]. Another phase III trial (CARES-310) [77] demonstrated that the combination of camrelizumab and rivoceranib was superior to sorafenib in terms of OS (HR, 0.62; *p* < 0.001) and PFS (HR, 0.52; *p* < 0.001) [Table 4].

**Table 4:** Overview of phase-3 clinical trials for systemic therapies in the advanced-stage HCC.

Trial name (Year)	Arms	OS, median (95%CI), months	HR of OS (95%CI)	PFS, median (95%CI), months	HR of PFS (95%CI)	ORR (%)	Any grade AE (%)
First-line							
SHARP (2008)	Sorafenib (n = 299)	10.7 (9.4–13.3)	0.69 (0.55–0.87)	5.5 (4.1–6.9)	0.58 (0.45–0.74)	2.0	80.0
	Placebo (n = 303)	7.9 (6.8–9.1)		2.8 (2.7–3.9)		1.0	52.0
REFLECT (2018)	Lenvatinib (n = 478)	13.6 (12.1–14.9)	0.92 (0.79–1.06)	7.4 (6.9–8.8)	0.66 (0.57–0.77)	18.8	99.0
	Sorafenib (n = 476)	12.3 (10.4–13.9)		3.7 (3.6–4.6)		6.5	99.0
IMbrave150 (2020)	Atezolizumab plus bevacizumab (n = 336)	19.2 (17.0–23.7)	0.66 (0.52–0.85)	6.9 (5.7–8.6)	0.65 (0.53–0.81)	27.3	86.0
	Sorafenib (n = 165)	13.4 (11.4–16.9)		4.3 (4.0–5.6)		11.9	95.0
ORIENT-32 (2021)	Sintilimab plus bevacizumab biosimilar (n = 380)	NR	0.57 (0.43–0.75)	4.6 (4.1–5.7)	0.56 (0.46–0.70)	21.0	99.0
	Sorafenib (n = 191)	10.4 (8.5-NR)		2.8 (2.7–3.2)		7.0	98.0
HIMALAYA (2022)	A: Tremelimumab plus durvalumab (n = 393)	16.4 (14.2–19.6)	A vs. C: 0.78 (0.65–0.92) B vs. C: 0.86 (0.73–1.03)	3.8 (3.7–5.3)	A vs. C: 0.90 (0.77–1.05) B vs. C: 1.02 (0.88–1.19)	20.1	97.4
	B: Durvalumab (n = 389)	16.6 (14.1–19.1)		3.7 (3.2–3.8)		17.0	88.9
	C: Sorafenib (n = 389)	13.8 (12.3–16.1)		4.1 (3.8–5.5)		5.1	95.5
RATIONALE-301 (2022)	Tislelizumab (n = 338)	15.9	0.85 (0.71–1.02)	2.2	1.1 (0.92–1.33)	14.3	96.2
	Sorafenib (n = 324)	14.1		3.6		5.4	100
COSMIC-312 (2022)	Cabozantinib plus atezolizumab (n = 432)	16.5 (96%CI 14.5–18.7)	0.98 (0.78–1.24)	6.9 (99%CI 5.7–8.2)	0.74 (0.56–0.97)	13.0	>99.0
	Sorafenib (n = 217)	15.5 (96%CI 12.2–20.0)		4.3 (99%CI 2.9–6.1)		4.6	99.0
	Cabozantinib (n = 188)			5.8 (99%CI 5.4–8.2)	0.78 (0.56–1.09)	7.4	99.0
LEAP-002 (2022)	Lenvatinib plus pembrolizumab (n = 395)	21.2 (19.0–23.6)	0.84 (0.71–0.99)	8.2 (6.4–8.4)	0.87 (0.73–1.02)	26.1	96.0
	Lenvatinib plus placebo (n = 399)	19.0 (17.2–21.7)		8.0 (6.3–8.2)		17.5	96.0
CARES-310 (2023)	Camrelizumab plus rivoceranib (n = 272)	22.1 (19.1–27.2)	0.62 (0.49–0.80)	5.6 (5.5–6.3)	0.52 (0.41–0.65)	25	97.0
	Sorafenib (n = 271)	15.2 (13.0–18.5)		3.7 (2.8–3.7)		6	93.0
**Second-line**							
RESORCE (2017)	Regorafenib (n = 379)	10.6 (9.1–12.1)	0.63 (0.50–0.79)	3.1 (2.8–4.2)	0.46 (0.37–0.56)	11.0	100
	Placebo (n = 194)	7.8 (6.3–8.8)		1.5 (1.4–1.6)		4.0	93.0
CELESTIAL (2018)	Cabozantinib (n = 470)	10.2 (9.1–12.0)	0.76 (0.63–0.92)	5.2 (4.0–5.5)	0.44 (0.36–0.52)	3.8	99.0
	Placebo (n = 237)	8.0 (6.8–9.4)		1.9 (1.9–1.9)		0.4	92.0
REACH-2 (2019)	Ramucirumab (n = 197)	8.5 (7.0–10.6)	0.71 (0.53–0.95)	2.8 (2.8–4.1)	0.45 (0.34–0.60)	4.6	97.0
	Placebo (n = 95)	7.3 (5.4–9.1)		1.6 (1.5–2.7)		1.1	86.3
KEYNOTE-240 (2020)	Pembrolizumab (n = 278)	13.9 (11.6–16.0)	0.78 (0.61–0.99)	3.0 (2.8–4.1)	0.72 (0.57–0.90)	18.3	96.4
	Placebo (n = 135)	10.6 (8.3–13.5)		2.8 (1.6–3.0)		4.4	90.3
CheckMate 040∗ (2020)	Ipilimumab plus nivolumab	22.8 (9.4-NR)	N/A	Not reported	Not reported	32	94.0

Abbreviations: AE: adverse effect; CI: confidence interval; HR: hazard ratio; NR: not-reached; ORR: objective response rate; OS: overall survival; PFS: progression-free survival ∗Phase 2 trial.

Current on-going phase 3 clinical trials for the advanced stage HCC were listed as [Table 5]. Recently, the interim analysis of a randomized CheckMate 9DW trial (NCT04039607) revealed that nivolumab plus ipilimumab demonstrated statistically significant median OS benefit (23.7 vs. 20.6 months; (HR, 0.79; *p* = 0.0180) as well as higher ORR (36% vs. 13%, *p* < 0.0001) and durable responses (30.4 vs. 12.9 months) with a manageable safety profile than lenvatinib or sorafenib for patients with uHCC. Apart from anti-PD-1/PD-L1 and anti-CTLA-4 antibodies, other ICIs for patients with unresectable HCC are currently under investigation. The phase III IMbrave152 trial (NCT05904886) is a two-arm study designed to evaluate PFS and OS between patients administered atezolizumab and those administered bevacizumab with or without tiragolumab, an anti-T-cell immunoglobulin and immunoreceptor tyrosine-based inhibitory motif domain (TIGIT) antibody. According to previous results of phase Ib/II MORPHEUS-liver study (NCT04524871) [78], addition of tiragolumab to atezolizumab plus bevacizumab resulted in higher ORR and longer PFS than those with atezolizumab plus bevacizumab alone, and no new safety signals were identified; thus, a promising novel first-line treatment option for patients with unresectable HCC was ascertained. The phase II RELATIVITY-106 trial (NCT05337137) [79] assessed the safety and efficacy of nivolumab plus bevacizumab with or without relatlimab, an anti-lymphocyte activation gene 3 (LAG3) antibody, in treatment-naïve patients with advanced/metastatic HCC [Table 5].

**Table 5:** Summary of active clinical trials of immune checkpoint inhibitor-based therapy in the advanced stage HCC.

Trial name (Registration No.)	Characteristics			End-point	Key findings
	Phase	Regimen	Arm		
First-line					
IMbrave152 (NCT05904886)	III	Atezolizumab plus bevacizumab, tiragolumab	**Arm 1**: Atezolizumab + bevacizumab + tiragolumab (anti-TIGIT) **Arm 2**: Atezolizumab + bevacizumab	OS, ORR, TTP, PFS, DOR, AE, QoL	–
ABE-LIVER (NCT05448677)	II	Atezolizumab plus bevacizumab, ezurpimtrostat	**Arm 1**: Atezolizumab + bevacizumab + Ezurpimtrostat (anti-PPT1) **Arm 2**: Atezolizumab + bevacizumab	PFS, ORR	–
LIVER-NET1 (NCT05546879)	I	Atezolizumab plus bevacizumab, NP137	Atezolizumab + bevacizumab + NP137 (anti-netrin-1)	AE, ORR, OS, PFS, QoL	–
RELATIVITY-106 (NCT05337137)	II	Nivolumab plus bevacizumab, relatlimab	**Arm 1**: Nivolumab + bevacizumab + relatlimab (anti-LAG3) **Arm 2**: Nivolumab + bevacizumab	ORR, PFS, OS, AE	–
CheckMate 9DW (NCT04039607)	III	Nivolumab plus ipilimumab, sorafenib/lenvatinib	**Arm 1**: Nivolumab + ipilimumab (n = 335) **Arm 2**: Sorafenib/lenvatinib (n = 333)	OS, ORR, DOR	OS: 23.7 vs. 20.6 mo (HR, 0.79; 95%CI, 0.65–0.96; p = 0.0180) ORR: 36% vs. 13% (p < 0.0001) DOR: 30.4 vs. 12.9 mo Any AE: 84% vs. 91% Grade 3/4 AE: 41% vs. 42%
SIERRA (NCT05883644)	III	Durvalumab plus tremelimumab	Durvalumab + tremelimumab (inferior liver function, poorer performance status, more advanced disease)	AE, ORR	–
TREMENDOUS (NCT05557838)	III	Durvalumab plus tremelimumab	Durvalumab + tremelimumab (Chinese patients)	OS, ORR, PFS, DOR, AE	–
GEMINI-HPB (NCT05775159)	II	Volrustomig, bevacizumab, lenvatinib, rilvegostomig	**Arm 1A**: Volrustomig (anti-CTLA-4/Anti-PD-1 bispecific antibody) **Arm 1B**: Volrustomig + bevacizumab **Arm 1C**: Volrustomig + lenvatinib **Arm 1D**: Volrustomig + rilvegostomig (anti-PD-1/anti-TIGIT bispecific antibody) + bevacizumab **Arm 1E**: Rilvegostomig + bevacizumab	ORR, AE, DOR, DCR, OS	–
**Second-line**					
IMbrave251 (NCT04770896)	III	Atezolizumab, lenvatinib, sorafenib	**Arm 1**: Atezolizumab + lenvatinib or sorafenib **Arm 2**: Lenvatinib or sorafenib	OS, PFS, ORR, TTP, DOR, AE	–
LIVERATION (NCT05201404)	III	Namodenoson	**Arm 1**: Namodenoson **Arm 2**: Placebo	OS, PFS, ORR	–
ATHENA (NCT06084884)	I/II	AZD5851	AZD5851 (CAR-T against GPC3) + lymphodepleting chemotherapy (fludarabine and cyclophosphamide)	AE, ORR, DOR, OS, PFS	–

Abbreviations: AE: adverse events; DCR: disease control rate; DOR: duration of response; DFS: disease free survival; ORR: objective response rate; OS: overall survival; PFS: progression-free survival; RFS: Recurrence-Free Survival; TTR: Time-to-Response.

### 5.2 Second-line and beyond therapies

In three phase III clinical trials, regorafenib (RESORCE trial) and cabozantinib (CELESTIAL trial), as well as the monoclonal antibody, ramucirumab (REACH-2 trial, when alpha-fetoprotein ≥400 ng/mL), are antiangiogenic targeted therapies that have been confirmed to improve OS in patients after failure of sorafenib treatment [[80], [81], [82]] [Table 4]. Although no further large randomized clinical trial was conducted to determine efficacy of any systemic therapy for patients whom failure of lenvatinib treatment, the above three mTKIs are already recommended by several international treatment guidelines [5,6,8,9]. Two small-scale retrospective studies [83,84] from the real-world data revealed the respective ORR and disease control rate (DCR) were 10.7–13.6% and 36.3–60.7% when treated with regorafenib for whom lenvatinib treatment failed. Another two small-scale retrospective studies [85,86] demonstrated the respective ORR and DCR were 0–3.8% and 42.3–80.0% when treated with ramucirumab for whom lenvatinib treatment failed. The efficacy of both two regimens were similar in the clinical trials after failure of sorafenib [80,82].

When considering the ICI therapy, two large clinical trials have revealed the efficacy and safety of the PD1 blockade, nivolumab (CheckMate 040 trial) and pembrolizumab (KEYNOTE-224 trial), in patients with advanced HCC and Child-Pugh class A liver function in whom failed after sorafenib treatment [87,88]. However, nivolumab monotherapy as the second-line treatment for advanced-stage HCC was opposed by the Food and Drug Administration of both the United States and Taiwan owing to its failure in the subsequent confirmatory CheckMate 459 trial [89]. Another clinical trial was conducted to test multiple regimens combining nivolumab plus ipilimumab, an anti-CTLA-4 antibody. All regimens demonstrated a satisfactory response rate at the expense of increased AE compared with nivolumab monotherapy [90]. The phase III KEYNOTE-240 trial revealed that pembrolizumab did not reach statistical significance in the OS and PFS when compared with placebo, possibly because of its overly aggressive statistical hypothesis. However, it still showed consistent tumor response rates, duration of response and survival benefit [91]. Moreover, another phase III clinical trial revealed that pembrolizumab provided significant prolonged survival benefits and efficacy especially for Asian patients with advanced HCC when compared with placebo [92]. In summary, either nivolumab plus ipilimumab or pembrolizumab monotherapy can be recommended for patients who could not tolerate or failed of current approved first-line mTKIs [5,11,44].

The clinical trials in progress determining the second-line treatment after failure of first-line ICI-based therapy included two phase III trials: IMbrave251 trial (NCT04770896), a two-arm study designed to evaluate the efficacy and safety of atezolizumab plus either lenvatinib or sorafenib versus lenvatinib or sorafenib alone in patients with advanced-stage HCC who have progressed on prior atezolizumab plus bevacizumab therapy; and LIVERATION trial (NCT05201404), a two-arm study to evaluate the efficacy and safety of namodenoson, A3 adenosine-receptor agonist, compared with placebo [Table 5].

### 5.3 Future study of advanced-stage HCC

In addition to ICIs, new immunotherapies that redirect T cells against tumor antigens through antibody fragments, without major histocompatibility complex presentation, have recently been investigated. In particular, bispecific antibodies (bsAbs) and chimeric antigen receptor-modified T-cell (CAR-T) therapy have demonstrated remarkable clinical responses in hematologic malignancies and have begun clinical evaluation for their use in HCC. bsAbs are antibody-based molecules with two different antigen-binding sites that can physically bridge together two different cells [93]. By simultaneously binding an antigen on tumor cells and a surface molecule on T cells, bsAbs can redirect and activate T cells to induce tumor lysis. From a structural perspective, bsAbs are categorized according to the presence or absence of the Fc region [94]. Bispecific T-Cell Engagers represent prototypical Fc-free bsAbs, with several new constructs under clinical evaluation for solid tumors now. Currently, several bsAbs are under both preclinical and clinical development for the treatment of various cancers [95], but little is known about their use in HCC. The phase II GEMINI-HPB (NCT05775159) trial evaluated the efficacy and safety of volrustomig (anti-CTLA-4/anti-PD-1 bsAb) or rilvegostomig (anti-PD-1/anti-TIGIT bsAb) as monotherapy and/or in combination with anticancer agents in patients with HCC [Table 5].

CAR-T is another therapeutic modality that has recently gained increasing attention. Using synthetic proteins encoded within an extracellular antigen recognition domain and an intracellular immune activation domain, CAR-T therapy targets tumor cells by stimulating the adaptive immune system of the host to recognize and eliminate tumor cells [96]. Currently, CAR-T cells have been used in various hematologic malignancies, such as acute lymphoblastic leukemia, and several clinical trials identifying potential CAR-T cell targets in HCC are ongoing [97]. The phase I/II ATHENA (NCT06084884) single-arm trial to evaluate the safety, cellular kinetics, and efficacy of AZD5851, a CAR-T therapy directed against glypican-3, as the second-line or beyond treatment of patients with advanced HCC is ongoing [Table 5].

## 6 Conclusions

We have witnessed the great progress in the systemic treatment of HCC over the past few decades. Currently, four oral mTKIs (sorafenib, lenvatinib, regorafenib, and cabozantinib), two antiangiogenic antibody (ramucirumab and bevacizumab), and six ICIs for treatment alone (pembrolizumab monotherapy) or in combination (ipilimumab plus nivolumab, atezolizumab plus bevacizumab and tremelimumab plus durvalumab) are licensed for use in Taiwan and other countries. An increasing number of ICIs, apart from anti-PD-1/PD-L1 and CTLA-4, such as anti-TIGIT and anti-LAG3, are under investigation and will soon gain approval for the treatment of unresectable HCC. In addition, the introduction of T-cell redirecting strategies, including bsAbs and CAR-T cells, has revolutionized the treatment landscape for specific hematological malignancies, extending their application to HCC. Future research should focus on an in-depth exploration of the mechanisms governing the establishment of tumor barriers and the intricate structural characteristics involved.

Notably, as therapeutic options expand and become effective, the stage-linked treatment decision-making algorithm may lead to undertreatment and suboptimal outcomes for patients with diseases beyond early- and intermediate-stage HCC. The refinement of the HCC treatment is needed, especially for those patients in the Asia-Pacific area. Novel evidence supports the use of aggressive treatment, particularly of multiple discipline therapy, for patients with major vessels invasion and a high tumor burden. Systemic therapy is no longer limited to advanced-stage HCC in the future.

## Declaration of competing interest

The authors have no conflicts of interest to declare.

## Funding

The authors did not receive any funding.
